# Dose-Dependent Effects of Branched-Chain Amino Acid Supplementation on Skeletal Muscle Morphology and Ultrastructure in Exercise-Trained Mice

**DOI:** 10.3390/nu18132124

**Published:** 2026-07-01

**Authors:** Yuhang Zhou, Xiaojuan Guo, Hai He, Yufei Yang, Yixin Zhang, Haiyue Feng, Zhiqiang Li

**Affiliations:** 1 Key Laboratory of Biotechnology and Bioengineering of State Ethnic Affairs Commission, Biomedical Research Center, School of Bioengineering, Northwest Minzu University, Lanzhou 730030, China; zhouyuhang200008@163.com (Y.Z.);; 2 Clinical Medical College, School of Dentistry, Gansu Medical University, Pingliang 744000, China; guoxiaojuan2025@163.com; 3 Key Laboratory of Oral Disease, School of Stomatology, Northwest Minzu University, Lanzhou 730030, China

**Keywords:** BCAA, muscle growth, mitochondria

## Abstract

Background: Branched-chain amino acids (BCAAs) regulate muscle protein metabolism, yet the systematic characterization of their dose-dependent morphological effects on exercised skeletal muscle remains limited. This study investigated the dose–response relationship between BCAA supplementation and skeletal muscle adaptations in exercise-trained mice. Methods: Seventy male Kunming mice were randomly assigned to seven groups (*n* = 10): a background group (no exercise), a control group (exercise + saline), and five exercise groups receiving BCAAs at 1–5 g/kg/day via intragastric gavage. Mice in the exercise groups performed 45 min of swimming daily (6 days/week) for 50 days. Gastrocnemius muscles were processed using hematoxylin–eosin staining, Masson trichrome staining, Gomori aldehyde fuchsin staining, and transmission electron microscopy. Data were analyzed using one-way ANOVA with Dunnett’s post hoc test. Results: BCAA supplementation increased gastrocnemius wet weight-to-body weight ratios and promoted denser fiber packing in a dose-dependent manner up to 3–4 g/kg/day. Deep-staining fiber proportion (putatively type II-like) increased progressively with BCAA concentration, plateauing at doses ≥ 3 g/kg/day, while elastic fiber content continued to rise through 5 g/kg/day. Mitochondrial size decreased as mitochondrial number increased; membrane and cristae thickness peaked at 3 g/kg/day. Sarcomere length, myofibril diameter, sarcoplasmic reticulum size, and transverse tubule diameter exhibited increasing trends. Conclusions: These findings establish a parameter-specific dose–response framework for BCAA-induced muscle remodeling. A supplemental dose of 3 g/kg/day above background dietary intake represents an effective threshold for maximizing indices of hypertrophic gains and mitochondrial structural maturation potentially indicative of functional enhancement. Higher doses (≥4 g/kg/day) elicited additional benefits in fiber density, mitochondrial proliferation, and elastic fiber content. Supplemental BCAA dosing strategies above constant background intake should be tailored to target specific structural outcomes, with functional validation required to confirm physiological relevance.

## 1. Introduction

Branched-chain amino acids (BCAAs) are essential nutrients that regulate muscle protein metabolism, exerting anti-fatigue effects, inhibiting muscle protein degradation, and promoting protein synthesis [1]. Under physiological conditions, mammalian skeletal muscle fiber number remains constant throughout adult life, with adaptive hypertrophy representing the primary mechanism of postnatal muscle enlargement [2]. Exercise potently stimulates this hypertrophic response, which is linked to activation of the mechanistic target of rapamycin complex (mTORC) pathway and enhanced protein synthesis [3,4]. During acute or chronic exercise, muscle cells sustain damage to varying degrees, prompting muscle satellite cells to mediate repair and contribute to hypertrophy [5,6].

Muscle proteins are primarily degraded during exercise and synthesized during rest, highlighting the importance of timely nutritional supplementation concurrent with training [7,8]. Although BCAAs have been shown to serve as direct or indirect regulators of muscle protein synthesis [1], the systematic morphological characterization of their dose-dependent effects on exercised skeletal muscle remains limited. Existing studies have largely focused on single-dose comparisons or binary outcomes (supplemented versus non-supplemented), leaving the dose–response landscape across multiple structural and functional parameters poorly defined. This gap is particularly relevant given that different muscle compartments—including contractile fibers, mitochondria, and extracellular matrix—may exhibit distinct sensitivities to BCAA availability, challenging the assumption that a single universal optimal dose exists for all adaptive outcomes.

While BCAA supplementation has been extensively studied in the context of muscle protein metabolism, the majority of animal studies have employed either single-dose designs or simple supplemented-versus-non-supplemented comparisons. For example, D’Antona et al. demonstrated that a single dose of BCAAs (1.5 g/kg/day in drinking water) promoted survival and mitochondrial biogenesis in middle-aged mice. Similarly, Banfi et al. [9] reported that the same single dose increased oxidative fiber content in dystrophic mdx mice. Yamanashi et al. [10] showed that a fixed concentration of 3% BCAAs in drinking water (~1.6 g/kg/day) ameliorated angiotensin II-induced muscle atrophy through mTOR activation. These studies established the biological efficacy of BCAAs in rodent models but did not address whether dose escalation within or beyond these ranges produces graded or parameter-specific adaptations.

Regarding mitochondrial remodeling, acute leucine administration has been shown to upregulate slow-fiber-related genes and PGC-1α expression in rat skeletal muscle [11], and chronic BCAA supplementation has been linked to increased mitochondrial content in aging mice. However, a systematic ultrastructural characterization of mitochondrial dose–response relationships—including membrane integrity, cristae architecture, and numerical density—has not been reported. Similarly, while BCAA-induced muscle hypertrophy is well documented at the gross level [12], the dose-dependent partitioning between hypertrophic versus hyperplastic remodeling pathways remains unexplored.

The present study was designed to address these gaps by investigating the dose-dependent morphological effects of BCAA supplementation on skeletal muscle in exercise-trained mice. Using a swimming exercise model, we examined gastrocnemius muscle adaptations across five supplemental BCAA doses (1–5 g/kg/day) spanning and exceeding the efficacious ranges reported in prior single-dose animal studies (1.5–1.6 g/kg/day) [10], and performed comprehensive analyses using routine histology, special staining, and transmission electron microscopy. We hypothesized that BCAA supplementation would induce concentration-dependent structural adaptations, exhibiting parameter-specific threshold patterns rather than a uniform dose ceiling across all measured indices.

## 2. Materials and Methods

### 2.1. Experimental Materials

#### 2.1.1. Experimental Reagents

The following reagents were used: 0.9% (*w*/*v*) sodium chloride solution (normal saline) (Sinopharm Chemical Reagent Co., Ltd., Shanghai, China); BCAA powder (leucine:isoleucine:valine = 4:1:1, *w*/*w*/*w*) (Zhejiang Yinuo Biotechnology Co., Ltd., Hangzhou, China); hematoxylin and eosin (HE) staining solution (Solarbio, Beijing, China); Masson’s trichrome staining kit (Solarbio, Beijing, China); Gomori’s aldehyde fuchsin staining solution (Solarbio, Beijing, China), and 4% paraformaldehyde (Sinopharm Group, Beijing, China).

#### 2.1.2. Experimental Instruments

Equipment included mouse gavage needles (No. 8), 1 mL syringes, 5 mL centrifuge tubes, frosted-end glass slides, cover slips, a precision balance (HC311; Shanghai Huachao Electric Appliance Co., Ltd., Shanghai, China), tissue embedding cassettes (Jinhua Yidi Medical Appliance Co., Ltd., Jinhua, China), an automatic tissue processor (YD-12P; Jinhua Yidi Medical Appliance Co., Ltd., Jinhua, China), an automatic embedding station (YD-6D; Jinhua Yidi Medical Appliance Co., Ltd., Jinhua, China), a rotary microtome (Leica Microsystems, Wetzlar, Germany), a slide warming table (Jinhua Yidi Medical Appliance Co., Ltd., Jinhua, China), a flotation bath (YD-AB; Jinhua Yidi Medical Appliance Co., Ltd., Jinhua, China), and a transmission electron microscope (TEM) (Hitachi High-Tech Corporation, Tokyo, Japan).

### 2.2. Experimental Methods

#### 2.2.1. Mice Feeding Experiments

The male Kunming (KM) mice used in this study were purchased from the Laboratory Animal Center, Lanzhou University (Lanzhou, China). Animals were SPF-grade, 8 weeks old, with body weights of 20–25 g. Seventy male KM mice (8 weeks old, 20–25 g body weight) were randomly assigned to seven groups (*n* = 10 per group) using a random number table. The sample size of *n* = 10 per group was determined based on comparable morphological studies of BCAA supplementation in rodent models [9,10,13], which have demonstrated statistically robust effects with similar group sizes. The groups comprised a background group (BG; no gavage, no exercise), a control group (CG; intragastric saline plus exercise), and five BCAA-supplemented groups receiving 1, 2, 3, 4, or 5 g/kg/day, designated as Groups 1–5. Mice were of specific-pathogen-free (SPF)-grade and were housed five per cage in standard polycarbonate cages (320 × 180 × 150 mm) under controlled conditions (12 h light/dark cycle, 22 ± 2 °C, 50 ± 5% humidity), with ad libitum access to standard chow and drinking water. Environmental enrichment was provided in the form of nesting material. Following arrival, mice were acclimatized to the facility for 7 days prior to the initiation of experimental procedures. The standard chow contained ≥18% crude protein and ≥4% crude fat, as reported by the manufacturer (Trophic Animal Feed High-Tech Co., Ltd., Nantong, China). A critical limitation of this design is that the exact BCAA and total amino acid composition of the standard chow were not analytically quantified. All groups received identical chow from the same batch, ensuring constant relative background dietary intake across the supplemental dose–response gradient. Consequently, the reported doses (1–5 g/kg/day) represent supplemental BCAA intake above an unquantified basal level, and the identified threshold of 3 g/kg/day should be interpreted as a relative supplemental dose rather than an absolute total BCAA requirement.

The selection of BCAA doses (1–5 g/kg/day) was based on the following rationale. First, previous studies on rodents have demonstrated biological efficacy of BCAA supplementation at ~1.5 g/kg/day via drinking water, which promoted mitochondrial biogenesis in skeletal muscle and improved survival in middle-aged mice [13]. Similarly, Banfi et al. [9] reported that the same dose increased oxidative fiber content in dystrophic mdx mice. Yamanashi et al. [10] showed that a fixed concentration of 3% BCAA in drinking water (~1.6 g/kg/day) ameliorated angiotensin II-induced muscle atrophy through mTOR activation. These studies established the biological efficacy of BCAAs in rodent models but employed single-dose designs. Second, subchronic toxicity studies reported no adverse effects at 2.5 g/kg/day for 3 months or 1.25 g/kg/day for 1 year, with a half-maximal lethal dose estimated at >10 g/kg [14]. Third, the no-observed-adverse-effect level (NOAEL) for leucine supplementation in rodents was identified at ~3.33–3.84 g/kg/day [15]. Accordingly, we selected 1, 2, and 3 g/kg/day to cover the established efficacy-to-safety range, and extended to 4 and 5 g/kg/day to characterize the dose–response relationship beyond the NOAEL threshold. This equidistant spacing (1 g/kg/day increments) was chosen to enable linear and quadratic polynomial contrast modeling of dose-dependent trends.

Swimming training was conducted in a cylindrical tank (50 cm diameter × 40 cm height) filled with water to a depth of 25 cm (exceeding mouse body length to prevent mice from supporting themselves with their tails), maintained at 30 ± 1 °C by a thermostatic system. Water was maintained under static conditions without artificial current to standardize exercise workload across all sessions. The training protocol consisted of 45 min/day, 6 days/week for 50 days. During the first week, swimming duration was progressively increased from 15 min/day to 45 min/day to allow acclimation. Water was changed daily, and mice were towel-dried and warmed under a heat lamp for 10 min after exercise to prevent hypothermia. Group allocation, data collection, and histological analysis were performed by investigators blinded to group assignments. Animal welfare was monitored daily using a standardized scoring system assessing body condition, activity level, and clinical signs (score range: 0–3 per category; cumulative score > 6 triggered veterinary review). Humane endpoints were predefined as: (i) body weight loss > 20% from baseline; (ii) inability to access food or water; (iii) severe locomotor impairment preventing swimming; (iv) cumulative welfare score > 8. No animals reached humane endpoint criteria or required intervention during the study. All animal experiments were conducted in accordance with the Animal Research: Reporting of In Vivo Experiments (ARRIVE) guidelines 2.0 [16] and were approved by the Animal Care Committee of Gansu Medical University (approval number 2025015; approval date: 7 October 2025). This study adhered to the 3R principles (Replacement, Reduction, Refinement) throughout.

#### 2.2.2. Morphological Experiment

Sampling and fixation. Thirty minutes after the final exercise session, mice were euthanized by cervical dislocation, and gastrocnemius muscles were rapidly excised following dissection. After weighing, tissue samples (5 mm × 1 mm) were excised parallel to the longitudinal axis of muscle fibers and fixed in 2.5% glutaraldehyde for transmission electron microscopic analysis. The remaining tissue was fixed in 4% paraformaldehyde, with the fixative replaced after 24 h, and then fixed for an additional 7 days. Additionally, liver and kidney tissues were collected from mice in each group and fixed in 4% paraformaldehyde for potential future analysis of BCAA side effects. However, histological processing and examination of these tissues were not completed within the scope of the present study.

Preparation of paraffin sections. Tissues were washed to remove fixative, dehydrated through a graded ethanol series, cleared in xylene, and embedded in paraffin. After solidification, blocks were stored at 4 °C for 24 h. Following conventional sectioning (5 μm thickness), slides were baked at 45 °C for 5 h and stored at 4 °C until use.

HE staining. Paraffin sections were dewaxed, rehydrated through a graded ethanol series (100%, 95%, 80%, and 70%, 5 min each), and stained with hematoxylin (5 min) and eosin (2 min). Muscle fiber cross-sectional area (CSA) and fiber number per unit area were quantified based on five random fields per section (400× magnification) using Image-Pro Plus 6.0 (Media Cybernetics, Inc., Rockville, MD, USA), calibrated with a stage micrometer prior to each measurement session. CSA was measured by manual tracing of individual fiber boundaries; fibers with incomplete cross-sections or oblique orientation were excluded. Fiber density was expressed as the number of fibers per mm^2^. All measurements were performed by two independent observers (inter-observer intraclass correlation coefficient (ICC) > 0.85), and the mean values were used for statistical analysis [17].

Masson staining. Paraffin sections were stained with Masson’s trichrome kit (Solarbio) according to the manufacturer’s protocol. Briefly, sections were stained with Ponceau red (0.5% in 1% acetic acid) for 5 min to label myofibrillar proteins, followed by phosphomolybdic acid differentiation (5 min) and aniline blue counterstaining (2 min) to label collagen. Under this protocol, muscle fibers with higher surrounding collagen content appear lighter due to aniline blue affinity for extracellular matrix, whereas fibers with denser myofibrillar packing exhibit deeper red-blue coloration due to Ponceau red retention. It should be noted that Masson’s trichrome staining does not directly identify myosin heavy chain (MyHC) isoforms and provides only indirect, morphology-based classification. Nuclei were stained black with Weigert’s iron hematoxylin. Fiber type classification was performed by two independent observers in a blinded manner; discrepancies were resolved by consensus. Five random fields per section (400× magnification) were captured, and the relative proportions of light-staining and deep-staining fibers were quantified using Image-Pro Plus 6.0 by setting consistent color threshold parameters across all sections [18].

Gomori staining. Elastic fibers in the connective tissue compartments of skeletal muscle (including perimysium, endomysium, and perivascular regions) were visualized using Gomori’s aldehyde fuchsin method (Solarbio). It is important to note that skeletal muscle fibers themselves do not contain elastic fibers; rather, elastic fibers are present in the surrounding connective tissue sheaths and blood vessels. Sections were oxidized in potassium permanganate (0.5%, 2 min), bleached in oxalic acid (2%, 1 min), stained in aldehyde fuchsin solution (20 min), and counterstained with Orange G (2 min). Under this protocol, elastic fibers in connective tissue appear purple due to aldehyde fuchsin affinity for elastin, whereas muscle fibers are decolorized to yellow using Orange G [19]. For quantitative analysis, five random fields per section (400× magnification) were captured. Elastic fiber content was expressed as the percentage of purple-stained area relative to total tissue area per field, measured using Image-Pro Plus 6.0 with a standardized hue–saturation–intensity (HSI) color threshold across all groups. Measurements were performed by an investigator blinded to group allocation.

#### 2.2.3. Image Analysis and Statistical Methods

The gastrocnemius-to-body weight ratio was calculated. For histological and ultrastructural analyses, a single section was selected from each mouse, and five random visual fields were captured per section as technical replicates. The mean value across these five fields was calculated for each mouse, and this mean was used as the data point for that individual animal. Thus, an individual mouse (*n* = 10 per group) was the experimental unit for all statistical comparisons.

For histological and ultrastructural analyses, five random fields were captured per section as technical replicates; the mean value across these five fields was calculated for each mouse and used for statistical analysis. Normality was assessed using the Shapiro–Wilk test. One-way analysis of variance (ANOVA) was performed to compare means across the seven groups. To characterize dose–response trends, orthogonal polynomial contrasts (linear and quadratic) were applied. Dunnett’s test was used for primary post hoc comparisons of each BCAA-supplemented group against the exercise control group (CG), as this procedure controls the family-wise error rate for multiple comparisons against a single control. Where differences among BCAA-supplemented groups were described in the Results Section, these were exploratory observations confirmed by Tukey’s honestly significant difference (HSD) test or pairwise t-tests with Bonferroni correction, as specified in the figure legends. For the precise *p*-values obtained from all post hoc comparisons, please contact the corresponding author. Statistical significance was set at *p* < 0.05. All analyses were performed using Statistical Package for the Social Sciences (SPSS) version 22.0 (IBM, Armonk, NY, USA).

## 3. Experimental Results

### 3.1. Characteristics Related to Mouse Growth Performance

**Figure 1:**
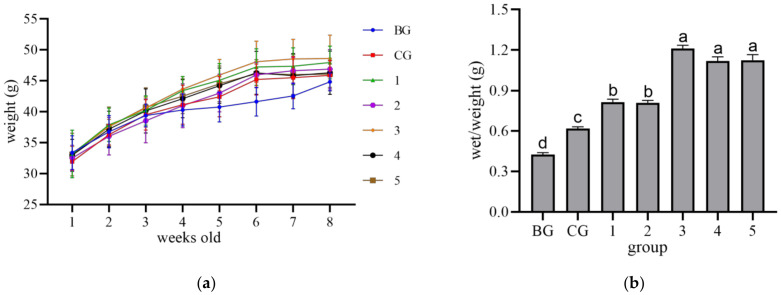
(**a**) Weekly body weight of mice across all groups over the 50-day experimental period. (**b**) Gastrocnemius/body weight. Data are represented as mean ± SD (*n* = 10 per group). Means without a common letter differ significantly (*p* < 0.05, one-way ANOVA with Dunnett’s post hoc test).

To assess the effects of different BCAA concentrations on growth performance in exercise-trained mice, body weight was monitored weekly throughout the 50-day experimental period (Figure 1a). All BCAA-supplemented groups exhibited greater weight gain than the BG, indicating that the swimming training protocol effectively stimulated overall growth. Among the exercise groups, BCAA-supplemented mice demonstrated higher weight gain than the CG, with the magnitude of gain appearing positively associated with BCAA concentration. Specifically, Groups 3 and 4 showed the most pronounced weight gain, whereas Group 5 showed weight gain comparable to Group 4, with no further significant increase.

The gastrocnemius wet weight-to-body weight ratio was significantly higher in the CG than in the BG (*p* < 0.05; Figure 1b), confirming that the 50-day swimming training induced a measurable hypertrophic effect in the hindlimb musculature. Compared with the CG, all BCAA-supplemented groups exhibited significantly increased gastrocnemius-to-body weight ratios (*p* < 0.05; Figure 1b). This effect exhibited a dose-dependent trend, with significantly higher ratios observed at increasing BCAA concentrations up to 4 g/kg/day (*p* < 0.05; Figure 1b). Notably, the ratio in Group 5 (5 g/kg/day) remained comparable to that in Group 4, suggesting that the dose–response relationship for muscle mass accretion plateaus beyond 4 g/kg/day under the present experimental conditions (*p* > 0.05; Figure 1b).

### 3.2. Routine Histology of Skeletal Muscle in Mice

#### 3.2.1. Conventional Histological Characteristics of Mouse Skeletal Muscle

**Figure 2:**
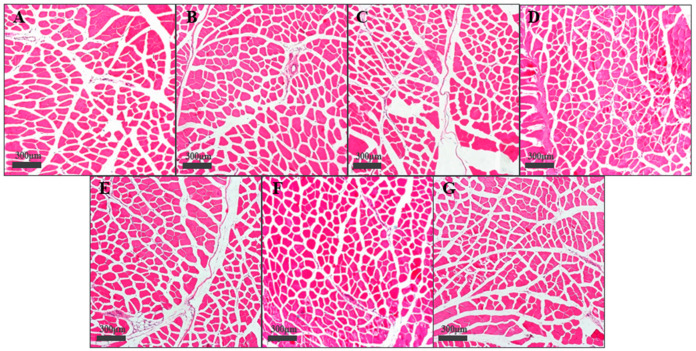
Histological structure of the mouse gastrocnemius muscle stained with hematoxylin and eosin (HE). (**A**) Background group (BG); (**B**) Control group (CG); (**C**–**G**) Experimental Groups 1–5 receiving BCAAs at 1, 2, 3, 4, and 5 g/kg/day, respectively. All images were captured at ×100 magnification with identical dimensions. Scale bar = 300 μm.

Hematoxylin and eosin staining revealed distinct architectural changes in gastrocnemius muscle organization across all experimental groups (Figure 2). In the BG, muscle fibers were loosely arranged with relatively wide inter-bundle spaces (Figure 2A), characteristic of sedentary muscle with minimal functional demand. Following exercise training (CG), fiber arrangement became moderately more compact (Figure 2B), with reduced inter-bundle spaces compared with the BG, indicating that swimming exercise alone induced structural remodeling. With increasing BCAA concentration, gastrocnemius muscle fibers progressively exhibited denser organization, with inter-bundle distances gradually decreasing through Groups 1–4 (Figure 2C–F). This progressive compaction suggests that BCAA supplementation enhanced the structural integrity of the muscle architecture beyond the effect of exercise alone. In Group 5, however, this trend showed a slight reversal (Figure 2G), with slightly increased inter-bundle spaces compared with Group 4, although this structural change was subtle and did not reach statistical significance in quantitative assessments.

#### 3.2.2. Conventional Histological Data of Skeletal Muscle in Mice

**Figure 3:**
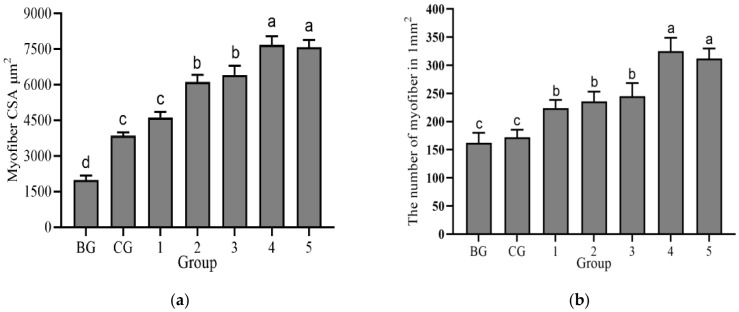
Quantitative histological analysis of gastrocnemius muscle. (**a**) Muscle fiber cross-sectional area (CSA). (**b**) Muscle fiber number per mm^2^. Data are represented as mean ± SD (*n* = 10 per group). Means without a common letter differ significantly (*p* < 0.05, one-way ANOVA with Dunnett’s post hoc test).

Muscle fiber cross-sectional area was significantly larger in the CG than in the BG (*p* < 0.05; Figure 3a), indicating that the swimming training protocol successfully induced exercise-dependent hypertrophy. Among the BCAA-supplemented groups, cross-sectional area was significantly increased in Groups 2–5 compared with the CG (*p* < 0.05; Figure 3a), whereas Group 1 (1 g/kg/day) did not differ significantly from the CG (*p* > 0.05; Figure 3a), suggesting that the lowest BCAA dose was insufficient to elicit measurable hypertrophic effects beyond exercise alone. The highest cross-sectional area values were observed in Groups 3 and 4. Group 3 (3 g/kg/day) showed particularly robust fiber enlargement. Group 5 showed a slight attenuation relative to Group 4, though this difference did not reach statistical significance (*p* > 0.05; Figure 3a), indicating that supraphysiological BCAA doses did not confer additional hypertrophic benefits and approached a ceiling effect for fiber cross-sectional enlargement.

Regarding muscle fiber number per square millimeter (Figure 3b), no significant difference was observed between the CG and the BG (*p* > 0.05; Figure 3b), indicating that exercise training alone did not substantially alter muscle fiber density in this model. All BCAA-supplemented groups exhibited significantly higher fiber density compared with both the BG and the CG (*p* < 0.05; Figure 3b), demonstrating that BCAA supplementation independently promoted increased fiber packing density. Within the BCAA groups, fiber number remained comparable among Groups 1–3 (*p* > 0.05; Figure 3b), whereas Groups 4 and 5 showed significant increases compared with Groups 1–3 (*p* < 0.05; Figure 3b). This divergent pattern, where fiber number continued to increase at higher doses while cross-sectional area plateaued, suggests that different structural parameters exhibit distinct dose–response sensitivities to BCAA supplementation. Group 5 displayed a slight decrease in fiber number compared with Group 4, but this difference was not statistically significant (*p* > 0.05; Figure 3b), suggesting that the upper limit for fiber density augmentation may be approached near 5 g/kg/day.

### 3.3. Special Histology of Mouse Skeletal Muscle

#### 3.3.1. Distribution Characteristics of Different Types of Muscle Fibers in Mouse Skeletal Muscle

**Figure 4:**
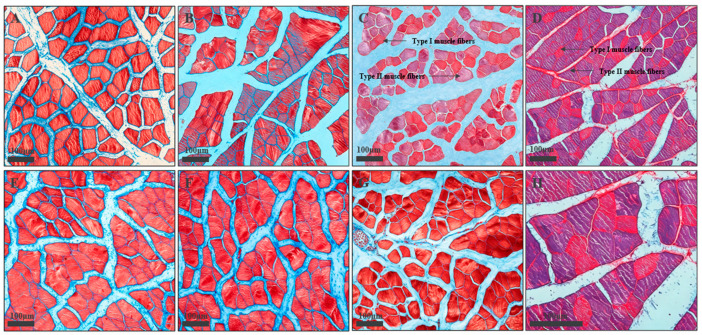
Distribution of type I and type II muscle fibers in the mouse gastrocnemius muscle stained with Masson’s trichrome. (**A**) Background group (BG); (**B**) Control group (CG); (**C**–**G**) Experimental Groups 1–5 receiving BCAAs at 1, 2, 3, 4, and 5 g/kg/day, respectively. (**H**) Enlargement of the boxed region in (**D**) (Group 2), showing classified muscle fibers at higher magnification. All images in (**A**–**G**) were captured at ×200 magnification with identical dimensions. Scale bar = 100 μm.

Masson staining revealed intermingled fibers with differential staining patterns: fibers with lighter staining (suggestive of higher perimysial collagen content, compatible with type I-like oxidative characteristics) and fibers with deeper red-blue staining (suggestive of denser myofibrillar content, compatible with type II-like glycolytic characteristics) (Figure 4). In the BG, light-staining fibers predominated, reflecting the postural and endurance characteristics of the gastrocnemius in sedentary mice (Figure 4A). Following exercise training (CG), a modest shift toward deep-staining fibers was evident, consistent with the known adaptive response of muscle to endurance exercise (Figure 4B). With increasing BCAA concentration, the ratio of deep-staining to light-staining fibers progressively increased through Groups 1–4 (Figure 4C–F), suggesting that BCAA supplementation may influence fiber composition patterns compatible with a glycolytic phenotype shift. In Group 5 (Figure 4G), this trend attenuated, exhibiting a distribution pattern comparable to that of Group 4, suggesting that the putative fiber type shift also exhibits a dose ceiling. The higher-magnification image (Figure 4H, enlargement of the boxed region in Figure 4D, Group 2) illustrates the morphological characteristics of deep-staining fibers (putatively type II-like) and light-staining fibers (putatively type I-like).

#### 3.3.2. Quantitative Analysis of Muscle Fiber Type Composition in Mouse Skeletal Muscle

**Figure 5:**
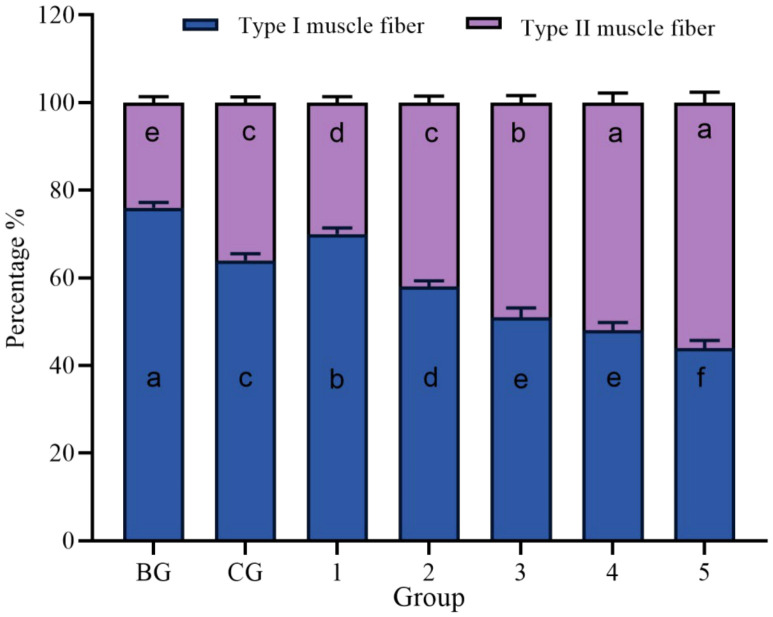
Distribution ratio of type I and type II muscle fibers in the mouse gastrocnemius muscle. Data are represented as mean ± SD (*n* = 10 per group). Means without a common letter above the type II fiber bars differ significantly (*p* < 0.05, one-way ANOVA with Dunnett’s post hoc test).

A quantitative assessment of fiber staining patterns revealed that in the BG, light-staining fibers (putatively corresponding to type I) accounted for 76.3% of the total fiber population, whereas deep-staining fibers (putatively corresponding to type II) comprised 23.7% (Figure 5). The CG showed a modest increase in type II fiber proportion compared with the BG, confirming that swimming exercise alone can induce modest fiber type remodeling (*p* > 0.05; Figure 5). In Group 1, fiber type distribution remained similar to that of the BG (*p* > 0.05; Figure 5), indicating that the lowest BCAA dose did not substantially influence fiber type composition beyond the effect of exercise. With increasing BCAA concentration, a progressive shift was observed: the proportion of type II fibers gradually increased, while that of type I fibers correspondingly decreased. Groups 3, 4, and 5 exhibited significantly higher proportions of type II fibers compared with the BG, the CG, and Groups 1 and 2 (*p* < 0.05; Figure 5). Importantly, no significant differences were detected among Groups 3, 4, and 5 (*p* > 0.05; Figure 5), demonstrating that the fiber type transition reaches a plateau at 3 g/kg/day and does not progress further at higher doses.

#### 3.3.3. Distribution Characteristics of Elastic Fibers in Skeletal Muscle of Mice

**Figure 6:**
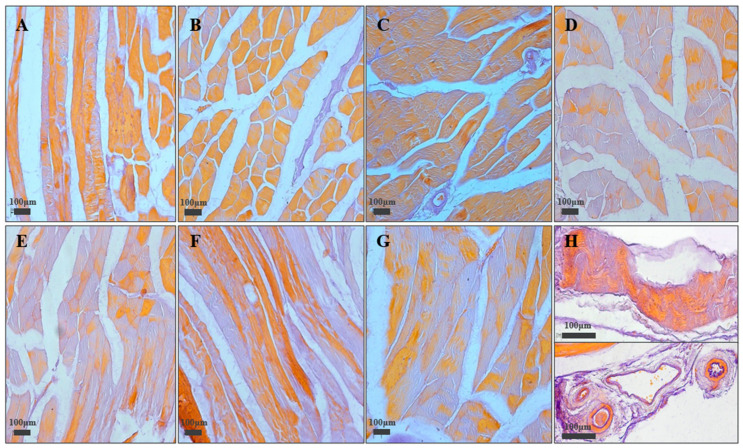
Distribution of elastic fibers in the mouse gastrocnemius muscle stained with Gomori’s aldehyde fuchsin. (**A**) Background group (BG); (**B**) Control group (CG); (**C**–**G**) Experimental Groups 1–5 receiving BCAAs at 1, 2, 3, 4, and 5 g/kg/day, respectively. (**H**) Enlargement of Group 3 (3 g/kg/day BCAAs) at ×400 magnification, showing the elastic fibers around and between muscles, while the elastic fibers around blood vessels and their periphery are shown below. All images in (**A**–**G**) were captured at ×200 magnification with identical dimensions. Scale bar = 100 μm.

Elastic fibers appeared as wavy structures interspersed within the perimysial and endomysial connective tissue and between muscle bundles. Skeletal muscle fibers themselves lack elastic fibers and were stained yellow by Orange G. Additionally, elastic fibers were observed surrounding blood vessels and within perivascular regions, consistent with the distribution presented by [20]. Representative images from each group are shown in Figure 6. In the BG, sparse elastic fibers were observed in the connective tissue of the gastrocnemius muscle (Figure 6A). Following exercise training (CG), a slight increase in perimysial and endomysial elastic fiber content was evident compared with the BG (Figure 6B). Among the BCAA-supplemented groups, connective tissue elastic fiber content progressively increased with rising BCAA concentration (Figure 6C–G). Notably, in the highest-dose group (Group 5), a tendency toward mild fibrosis was observed, suggesting a potential adverse effect at supraphysiological doses. The higher magnification image (Figure 6H, enlargement of Group 3 at ×400) illustrates the morphological characteristics of elastic fibers surrounding muscle tissue and blood vessels.

#### 3.3.4. Quantitative Analysis of Elastic Fiber Content

**Figure 7:**
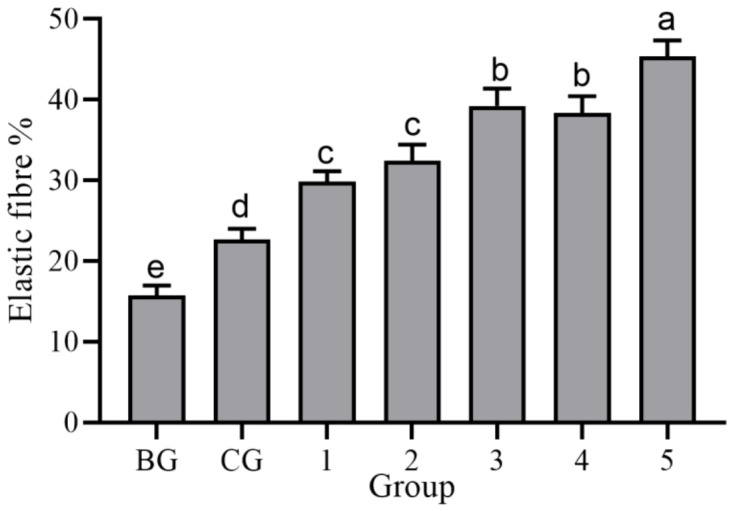
Proportion of elastic fibers in the gastrocnemius muscle of mice. Data are represented as mean ± SD (*n* = 10 per group). Means without a common letter differ significantly (*p* < 0.05, one-way ANOVA with Dunnett’s post hoc test).

Quantitative analysis confirmed that elastic fiber content in the CG was significantly higher than in the BG (*p* < 0.05; Figure 7), indicating that swimming exercise alone stimulated elastic fiber deposition. Among the BCAA-supplemented groups, elastic fiber content further increased in a dose-dependent manner, with progressively higher proportions observed as BCAA concentration increased from 1 to 5 g/kg/day. Group 5 exhibited the highest elastic fiber content among all groups, and was significantly different from all other groups (*p* < 0.05; Figure 7). This continued increase in elastic fiber content at the highest dose, contrasting with the plateau observed for fiber type distribution and cross-sectional area, demonstrates that different structural parameters exhibit distinct dose–response relationships to BCAA supplementation.

### 3.4. Ultrastructural Characteristics of Skeletal Muscle in Mice

#### 3.4.1. Ultrastructural Characteristics of Mouse Skeletal Muscle Mitochondria

**Figure 8:**
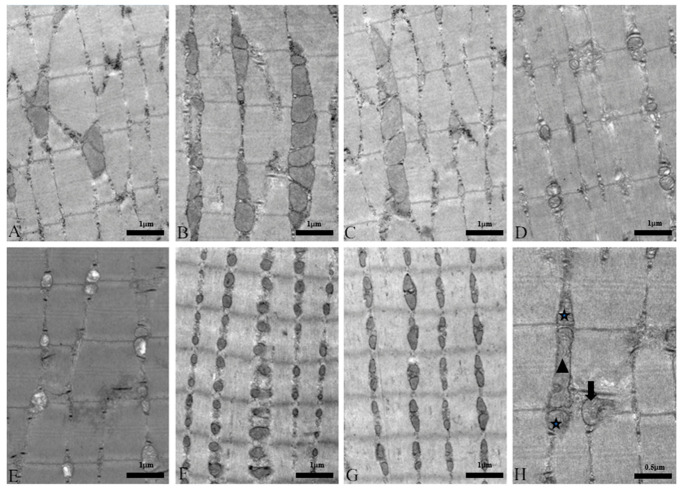
Mitochondrial ultrastructure in the gastrocnemius muscle of mice. (**A**) Background group (BG); (**B**) Control group (CG); (**C**–**G**) Experimental Groups 1–5 receiving BCAAs at 1, 2, 3, 4, and 5 g/kg/day, respectively. (**H**) Enlargement of Group 3 (3 g/kg/day BCAAs) at ×8000 magnification, showing the neuromuscular junction. ↓, synapse; ★, mitochondrion; ▲, sarcoplasmic reticulum; black lumen, transverse tubule. All images in (**A**–**G**) were captured at ×8000 magnification with identical dimensions. Scale bar = (**A**–**G**) 1 μm; (**H**) 0.5 μm.

Transmission electron microscopy results revealed detailed mitochondrial architecture and subcellular organization in gastrocnemius muscle (Figure 8). Mitochondria were located between myofibrils, distributed longitudinally along the myofibrillar axis. Neuromuscular junctions, sarcoplasmic reticulum, and transverse tubules were also visualized. In the BG, mitochondria were sparsely distributed (Figure 8A), with relatively small individual mitochondrial profiles. Following exercise training (CG), mitochondria appeared more abundant and larger (Figure 8B), with a relatively continuous distribution pattern along the myofibrils, consistent with the well-documented mitochondrial biogenic response to endurance exercise. In Group 1 (Figure 8C), mitochondrial volume decreased markedly compared with the CG, whereas mitochondrial number began to increase, suggesting an early adaptive response to BCAAs involving mitochondrial fragmentation or fission. With increasing BCAA concentration, mitochondrial size gradually recovered toward BG levels, while mitochondrial number exhibited a clear dose-dependent increase (Figure 8C–G), indicating that BCAA supplementation promoted mitochondrial proliferation rather than individual mitochondrial hypertrophy. The higher magnification image (Figure 8H, enlargement of Group 3 at ×8000) illustrates the ultrastructural features of the neuromuscular junction, including synapses (↓), mitochondria (★), sarcoplasmic reticulum (▲), and transverse tubules (black lumen).

#### 3.4.2. Mitochondrial Data of Skeletal Muscle in Mice

**Figure 9:**
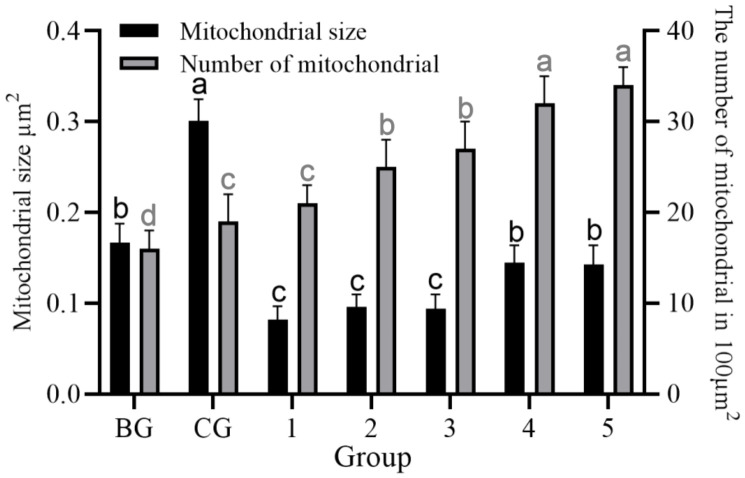
Quantitative analysis of mitochondrial morphology in mouse gastrocnemius muscle. Mitochondrial cross-sectional area (as a proxy for mitochondrial size); mitochondrial number per 100 μm^2^. Data are represented as mean ± SD (*n* = 10 per group). Means without a common letter differ significantly (*p* < 0.05, one-way ANOVA with Dunnett’s post hoc test).

The quantitative analysis of mitochondrial size and number per 100 μm^2^ confirmed these visual observations (Figure 9). The CG exhibited significantly increased mitochondrial size and number compared with the BG (*p* < 0.05; Figure 9), confirming the exercise-induced enhancement of mitochondrial content. In Group 1, mitochondrial volume decreased rapidly compared with the CG (*p* < 0.05; Figure 9), reaching levels below those of the CG. With increasing BCAA concentration, mitochondrial size gradually recovered to levels comparable to those of the BG (*p* > 0.05; Figure 9), demonstrating that higher BCAA doses normalized mitochondrial volume. Conversely, mitochondrial number exhibited a dose-dependent increase across Groups 1–5, with Group 5 showing the highest mitochondrial count among all groups (*p* < 0.05; Figure 9). This divergent pattern in which mitochondrial size normalized while number continued to increase suggests that BCAA supplementation shifts mitochondrial adaptation from hypertrophic to hyperplastic mechanisms.

**Figure 10:**
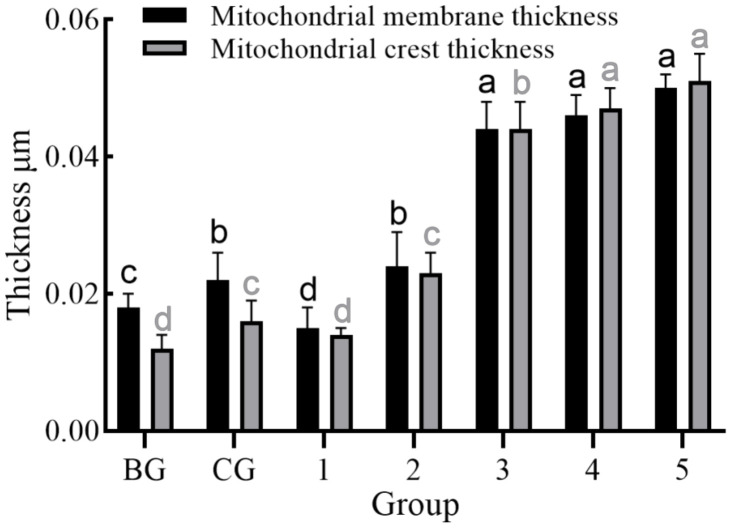
Mitochondrial membrane and cristae thickness in mouse gastrocnemius muscle. Data are represented as mean ± SD (*n* = 10 per group). Means without a common letter differ significantly (*p* < 0.05, one-way ANOVA with Dunnett’s post hoc test).

Mitochondrial membrane thickness and cristae thickness were also quantified to assess functional integrity (Figure 10). The CG exhibited significantly increased membrane thickness compared with the BG *(p* < 0.05; Figure 10); however, cristae thickness in the CG was markedly lower than that of the membrane, indicating an uncoupled structural response to exercise alone. In Group 1, both parameters decreased compared with the CG (*p* < 0.05; Figure 10), suggesting that low-dose BCAAs may transiently disrupt mitochondrial membrane integrity. With increasing BCAA concentration, both membrane and cristae thickness progressively increased, with the most pronounced values observed in Group 3 (*p* < 0.05; Figure 10). In contrast to the CG, the BCAA-supplemented groups exhibited synchronous increases in membrane and cristae thickness, maintaining a relatively proportional relationship (*p* < 0.05; Figure 10), suggesting that BCAAs promote coordinated structural maturation of mitochondrial membranes.

#### 3.4.3. Ultrastructural Characteristics of Myofibrils and Sarcoplasmic Reticulum in Mouse Skeletal Muscle

**Figure 11:**
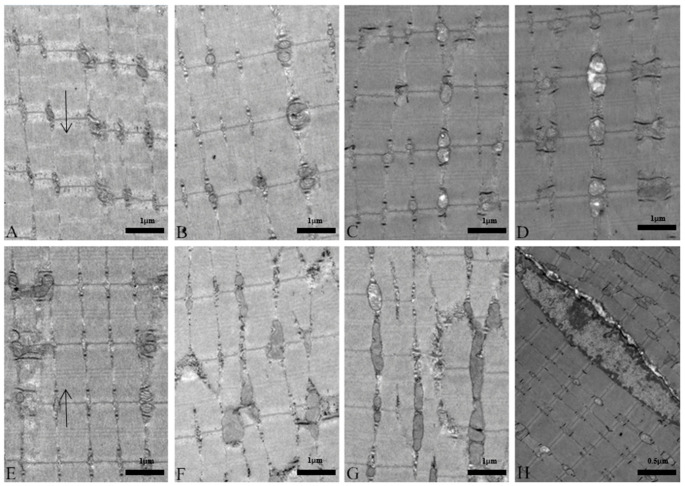
Ultrastructure of myofibrils and sarcoplasmic reticulum in mouse gastrocnemius muscle. (**A**) Background group (BG); (**B**) Control group (CG); (**C**–**G**) Experimental Groups 1–5 receiving BCAAs at 1, 2, 3, 4, and 5 g/kg/day, respectively. (**H**) Enlargement of Group 3 (3 g/kg/day BCAAs) at ×8000 magnification, showing the nuclear structure within the tissue. ↑, H-band and M-line; ↓, I-band and Z-line. All images in (**A**–**G**) were captured at ×8000 magnification with identical dimensions. Scale bar = (**A**–**G**) 1 μm; (**H**) 0.5 μm.

Transmission electron microscopy results revealed alternately distributed light and dark bands in sarcomeres, with mitochondria and sarcoplasmic reticulum located between the Z-lines (Figure 11). Transverse tubules were oriented parallel to the Z-lines, while the sarcomere axis was perpendicular to these structures. Electron-dense granular deposits, consistent with calcium precipitates, were observed in some regions. In the BG, myofibrils exhibited regular organization with distinct sarcomere boundaries (Figure 11A). Following exercise training (CG), structural modifications were evident, including subtle alterations in myofibril width and sarcomere length (Figure 11B). Among the BCAA-supplemented groups (Figure 11C–G), variations in myofibril width and sarcomere length were observed across different concentrations, with progressive structural enhancement through the mid-range doses. The higher magnification image (Figure 11H, enlargement of Group 3 at ×8000) illustrates the nuclear structure within the tissue, with arrows indicating the H-band and M-line (↑), and the I-band and Z-line (↓).

#### 3.4.4. Data on Myofibrils and Sarcoplasmic Reticulum of Mouse Skeletal Muscle

**Figure 12:**
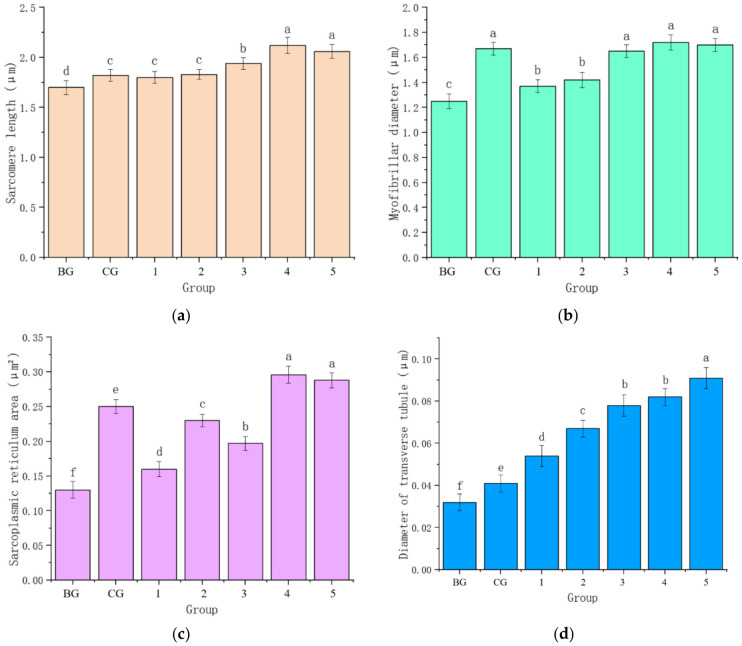
Quantitative ultrastructural analysis of myofibrils and sarcoplasmic reticulum in mouse gastrocnemius muscle. (**a**) Sarcomere length. (**b**) Myofibril diameter. (**c**) Sarcoplasmic reticulum size. (**d**) Transverse tubule diameter. Data are represented as mean ± SD (*n* = 10 per group). Means without a common letter differ significantly (*p* < 0.05, one-way ANOVA with Dunnett’s post hoc test).

Sarcomere length (Figure 12a), myofibril diameter (Figure 12b), sarcoplasmic reticulum size (Figure 12c), and transverse tubule diameter (Figure 12d) were quantitatively analyzed. Myofibril diameter, sarcoplasmic reticulum size, and transverse tubule diameter were significantly larger in the CG than in the BG (*p* < 0.05; Figure 12b–d), indicating that swimming exercise alone induced substantial ultrastructural enhancement. Among the BCAA-supplemented groups, these three parameters (Figure 12b–d) showed progressive increases with rising BCAA concentration (*p* < 0.05; Figure 12b–d). This dose-dependent trend was particularly evident in myofibril diameter (Figure 12b) and sarcoplasmic reticulum size (Figure 12c), which exhibited the most pronounced increases among the measured ultrastructural parameters. In Group 5, all three parameters (Figure 12b–d) showed slight decreases compared with Group 4, though these differences did not reach statistical significance (*p* > 0.05; Figure 12b–d), suggesting that the ultrastructural enhancement of contractile and calcium-handling apparatus also approaches a ceiling at supraphysiological BCAA doses.

Regarding sarcomere length (Figure 12a), the CG exhibited significantly longer sarcomeres compared with the BG (*p* < 0.05; Figure 12a), confirming that exercise induced sarcomeric elongation. Among the BCAA-supplemented groups, sarcomere length remained relatively unchanged in Groups 1 and 2 compared with the CG (*p* > 0.05; Figure 12a), indicating that lower BCAA doses did not substantially influence sarcomere length beyond the effect of exercise. In Groups 3 and 4, sarcomere length increased further (*p* < 0.05; Figure 12a), with Group 3 showing particularly notable elongation. Group 5 showed a slight decrease in sarcomere length compared with Group 4, though this difference was not statistically significant (*p* > 0.05; Figure 12a). This pattern in which sarcomere length peaks at mid-range doses while other parameters continue to increase further demonstrates the parameter-specific nature of BCAA dose–response relationships.

## 4. Discussion

### 4.1. Dose-Dependent Muscle Hypertrophy and the Threshold Concept

The present study systematically characterized the dose-dependent morphological adaptations of BCAA supplementation in the skeletal muscle of exercise-trained mice. Rather than identifying a single universal optimal dose, our findings reveal a parameter-specific dose–response framework in which different structural and ultrastructural parameters exhibit distinct threshold and plateau patterns. BCAA supplementation at doses ranging from 1 to 5 g/kg/day augmented gastrocnemius muscle mass, fiber density, and contractile apparatus ultrastructure in exercised mice; however, the magnitude and pattern of these adaptations varied considerably across parameters. These results support the concept that BCAA-induced muscle remodeling is not governed by a uniform dose-effect relationship but instead involves differential sensitivities of hypertrophic, hyperplastic, and connective tissue responses.

The observed increase in gastrocnemius wet weight and fiber cross-sectional area following swimming training is consistent with established evidence that endurance exercise stimulates skeletal muscle hypertrophy [21,22]. Under standardized conditions, muscle tissue weight is relatively stable within species but can be markedly influenced by training, nutritional status, and hormonal factors during development and maturity [22,23,24]. Ni et al. [25] reported that the age-related weight reduction trend of gastrocnemius muscle in model mice was markedly attenuated by exercise training compared with sedentary controls, further supporting the protective and growth-promoting effects of swimming exercise on hindlimb musculature. The present findings demonstrate that BCAA supplementation further augmented exercise-induced muscle mass accretion in a dose-dependent manner up to 3–4 g/kg/day, with the highest wet weight-to-body weight ratios observed in Groups 3 and 4. Notably, Group 5 did not exhibit further significant gains in muscle mass or fiber cross-sectional area compared with Group 4, suggesting that the hypertrophic response to BCAAs reaches a ceiling at approximately 3–4 g/kg/day under the present experimental conditions. This plateau may reflect saturation of BCAA uptake or utilization mechanisms in muscle tissue. Excessive amino acid availability can induce insulin resistance or nutrient overload, thereby preventing further anabolic stimulation [15,26]. Xu et al. [27] reported that BCAAs promote protein synthesis. While our morphological data are compatible with this mechanism, direct measurement of protein synthesis markers (e.g., puromycin incorporation, S6 phosphorylation) was not performed. The divergence between muscle mass and fiber density in which fiber number continued to increase through Group 5 while cross-sectional area plateaued suggests that BCAA supplementation may differentially influence hyperplastic versus hypertrophic remodeling pathways.

### 4.2. Fiber Type Plasticity and Connective Tissue Remodeling

In addition to promoting muscle mass accretion, BCAA supplementation induced significant changes in muscle fiber staining patterns compatible with fiber composition remodeling. The progressive increase in deep-staining fiber proportion (putatively type II-like) from Group 1 through Group 4, with a plateau among Groups 3–5, suggests that BCAA availability may influence putative fiber type plasticity. However, because Masson’s trichrome staining does not directly identify myosin heavy chain isoforms, these observations represent staining patterns compatible with fiber composition changes rather than definitive evidence of fiber type transition. Deep-staining fibers are enriched in myofibrillar proteins and exhibit high glycolytic capacity, making them well suited for high-intensity, explosive exercise [28]. If validated by MyHC immunohistochemistry, the preferential increase in putative type II-like fiber content with BCAA supplementation may contribute to improved resistance to exercise-induced fatigue, as these fibers possess superior anaerobic capacity compared with light-staining fibers [1]. This interpretation is based solely on morphological staining patterns, as direct contractile protein isoform identification and functional validation of fatigue resistance were not performed.

Regarding connective tissue adaptations, both exercise and BCAA supplementation increased elastic fiber content, with the highest values observed in Group 5. Elastic fibers, together with myofibrils, constitute the primary structural components responsible for passive muscle recoil [29,30]. The continued increase in elastic fiber content at 5 g/kg/day—contrasting with the plateau observed for fiber type distribution—further underscores the parameter-specific nature of BCAA dose responses. However, the tendency toward increased extracellular matrix deposition in Group 5 warrants caution, as excessive connective tissue accumulation may eventually impair active contractile function. This dichotomy between fiber type plasticity and connective tissue remodeling illustrates that different muscle compartments exhibit distinct dose sensitivities, supporting the need for parameter-specific rather than universal dosing strategies.

### 4.3. Distribution of Elastic Fibers in Skeletal Muscle Connective Tissue

The presence of elastic fibers in skeletal muscle warrants clarification. While individual muscle fibers lack elastic fibers, the connective tissue sheaths surrounding them—namely the epimysium, perimysium, and endomysium—do contain elastin. Kovanen and Suominen reported that elastin constitutes approximately 0.4% of muscle dry weight, localized primarily in the connective tissue compartment [20]. More recently, TEM studies have directly visualized elastic fibers in both the endomysium and perimysium of human skeletal muscle [31].

In the present study, Gomori’s aldehyde fuchsin staining revealed connective tissue elastic fibers (purple) interspersed between muscle fibers (yellow) and surrounding blood vessels. The observed progressive increase in perimysial and endomysial elastic fiber content with BCAA supplementation suggests enhanced connective tissue remodeling. However, we acknowledge that the functional significance of increased connective tissue elastin in muscle adaptation remains unclear, and whether this contributes to improved passive elasticity or merely reflects fibrotic tendencies requires further investigation.

### 4.4. Mitochondrial Biogenesis and Functional Adaptation

Ultrastructural analysis revealed distinct mitochondrial adaptations that diverged from the hypertrophic patterns observed at the tissue level. Exercise training alone induced mitochondrial enlargement and increased cristae spacing, consistent with physiological compensatory mechanisms to enhance oxidative phosphorylation under increased energy demand [32,33]. Under extreme conditions such as toxicity, hypoxia, or tissue damage, mitochondria may disintegrate and decrease in number, which represents pathological rather than adaptive responses [34]. In the present study, however, the observed mitochondrial changes reflected adaptive remodeling rather than pathological deterioration, as evidenced by the coordinated increase in mitochondrial number and membrane integrity in BCAA-supplemented groups.

The observed mitochondrial proliferation is morphologically reminiscent of mitochondrial biogenesis, a process classically associated with PGC-1α-dependent mechanisms [35,36]. However, without direct measurement of PGC-1α expression, NRF-1, TFAM, or mitochondrial DNA content, this interpretation remains speculative. It has been proposed that structural modifications of mitochondrial membranes and cristae may facilitate biochemical reactions and functional protein synthesis [37]. While our observation of synchronous membrane and cristae thickening is morphologically compatible with this hypothesis, direct evidence of enhanced respiratory chain activity or ATP synthesis was not obtained. We speculate that, by serving as direct energy substrates, BCAAs might reduce the compensatory demand on mitochondrial ATP production, potentially allowing mitochondrial volume to decrease while preserving oxidative capacity through an increased organelle number. This hypothesis requires validation by respirometry or ATP production assays.

### 4.5. Excitation–Contraction Coupling and Ultrastructural Remodeling

The observed increases in myofibril diameter, sarcoplasmic reticulum size, and transverse tubule diameter in BCAA-supplemented mice indicate substantial remodeling of the excitation–contraction coupling apparatus. Sarcomere length increased slightly with exercise and showed further augmentation at mid-range BCAA doses, whereas myofibril diameter exhibited a biphasic response with an initial decrease followed by recovery. These differential effects may reflect distinct influences of BCAA on actin and myosin synthesis, with BCAA availability exerting a greater stimulatory effect on sarcomeric elongation than on radial growth. The progressive expansion of SR and transverse tubules provides the structural prerequisite for potentially enhanced calcium handling. Whether this structural expansion translates into improved calcium kinetics requires direct functional measurement around muscle cells [38,39]. If functionally competent, larger SR volumes might accommodate greater calcium reserves, potentially improving resistance to ion depletion and delay the onset of muscular fatigue. The slight attenuation of these ultrastructural parameters in Group 5, although not statistically significant, suggests that the excitation–contraction coupling apparatus may also approach a structural ceiling at supraphysiological BCAA doses.

### 4.6. Strengths and Limitations

This study has several strengths. We established a dose–response framework with five BCAA concentrations (1–5 g/kg/day) and appropriate controls, enabling parameter-specific threshold characterization. Multimodal morphological assessment (HE, Masson, Gomori, and TEM) provided integrated insights from tissue to organelle levels. Blinded investigators performed quantitative analyses with verified reliability (ICC > 0.85). Orthogonal polynomial contrasts with Dunnett’s test appropriately modeled dose–response trends while controlling family-wise error. The 50-day standardized swimming protocol with progressive acclimation ensured reproducible stimuli.

Despite these strengths, several methodological limitations should be acknowledged. First, muscle fiber typing was performed using MyHC immunohistochemistry, which directly identifies MyHC I isoform expression and provides a definitive classification of type I versus type II fibers. This method represents a significant improvement over the initial Masson’s trichrome approach, which does not distinguish MyHC isoforms. However, MyHC immunohistochemistry with a single antibody (anti-MyHC I) does not distinguish between type II subtypes (IIa, IIx, IIb). Future studies should employ additional MyHC antibodies (anti-IIa, anti-IIx) or Adenosine triphosphatase (ATPase) histochemistry at multiple pH preincubations to fully characterize fiber type transitions. Second, the quantitative analysis of elastic fibers was based on two-dimensional area fraction, which may not fully capture the three-dimensional complexity of extracellular matrix architecture. Third, functional performance measures such as grip strength, swimming exhaustion time, and blood biochemical markers were not assessed; therefore, the observed morphological adaptations cannot be directly extrapolated to exercise capacity or fatigue resistance. Fourth, a fundamental limitation is that the exact BCAA and total protein contents of the standard chow were not analytically quantified. This study specifically examined the dose–response to supplemental BCAAs above constant background dietary intake. While the relative comparison between supplemental doses remains internally valid because all groups received identical chow from the same batch, the absolute total BCAA intake (dietary plus supplemental) could not be determined. Consequently, the 3 g/kg/day threshold should be strictly interpreted as the optimal supplemental dose above unquantified background intake under the present experimental conditions, not as an absolute or generally applicable optimal dose. Future studies should employ defined diets (e.g., AIN-93G) with precisely controlled amino acid composition to establish absolute requirements and validate the thresholds identified here. Fifth, the use of orthogonal polynomial contrasts and Dunnett’s test represents an improvement over conventional pairwise comparisons for dose–response characterization; however, the cross-sectional nature of the data limits causal inferences regarding the temporal progression of BCAA-induced adaptations. Sixth, the swimming exercise model, although well characterized, may not fully replicate the mechanical loading patterns of resistance training; thus, the generalizability of these findings to other exercise modalities requires further investigation. Seventh, although liver and kidney tissues were collected and fixed, histological analysis of these organs was not performed. Consequently, potential systemic adverse effects of high-dose BCAA supplementation could not be assessed. The observation of a tendency toward mild fibrosis in skeletal muscle of Group 5 highlights the need for comprehensive safety evaluation, including hepatic and renal histopathology as well as serum biochemical markers, in future studies. Eighth, the elastic fibers quantified in this study were localized to the connective tissue sheaths (perimysium, endomysium) and perivascular regions, not within muscle fibers themselves. Skeletal muscle fibers lack elastic fibers; their passive elasticity is primarily conferred by the giant protein titin. The observed increase in ‘elastic fiber content’ with BCAA supplementation should be interpreted as connective tissue remodeling rather than alterations in muscle fiber intrinsic elasticity.

## 5. Conclusions

In conclusion, this study establishes a parameter-specific supplemental dose–response framework for BCAA-induced skeletal muscle remodeling in exercise-trained mice. Under the specific conditions of standard chow plus graded intragastric supplementation, a supplemental dose of 3 g/kg/day above background intake represents an effective threshold for robust morphological adaptations, encompassing maximal indices of hypertrophy, coordinated mitochondrial membrane structural maturation, and ultrastructural features suggestive of enhanced excitation–contraction coupling. Supraphysiological supplemental doses (≥4 g/kg/day) demonstrated continued benefits in selected parameters, including muscle fiber density, mitochondrial number, and elastic fiber content, but exhibited plateauing or non-significant attenuation in muscle mass and fiber cross-sectional area. These findings indicate that supplemental BCAA-induced muscle remodeling involves differential dose sensitivities across distinct structural compartments, challenging the concept of a single universal optimal dose.

This study specifically examined the dose–response to supplemental BCAAs above constant background dietary intake, rather than absolute total BCAA requirements. The reported threshold should be interpreted as a relative supplemental dose under the present experimental conditions and should not be generalized as an absolute or universally applicable optimal dose without validation in defined dietary formulations. Future research should integrate functional performance measures with defined dietary formulations to refine parameter-specific BCAA dosing strategies and enhance the translational relevance of these morphological observations. Direct functional measurements—including grip strength, contractile force, and mitochondrial respiration—are warranted in future studies to validate the physiological significance of these morphological observations.

## Data Availability

The data that support the findings of this study are available from the corresponding authors upon reasonable request. No protocol was registered for this study.

## Abbreviations

The following abbreviations are used in this manuscript:

ANOVA	Analysis of variance
ARRIVE	Animal Research: Reporting of In Vivo Experiments
ATP	Adenosine triphosphate
ATPase	Adenosine triphosphatase
BCAAs	Branched-chain amino acids
BG	Background group
CG	Control group
CSA	Cross-sectional area
HE	Hematoxylin and eosin
HSI	Hue–saturation–intensity
ICC	Intraclass correlation coefficient
KM	Kunming (mouse strain)
mTORC	Mechanistic target of rapamycin complex
MyHC	Myosin heavy chain
PGC-1α	Peroxisome proliferator-activated receptor gamma coactivator 1-alpha
SD	Standard deviation
SPSS	Statistical Package for the Social Sciences
TEM	Transmission electron microscopy
